# Association between functional disability with postural balance among patients with chronic low back pain

**DOI:** 10.3389/fneur.2023.1136137

**Published:** 2023-05-23

**Authors:** Pingge Sun, Kunbin Li, Xianli Yao, Zhiyuan Wu, Yafei Yang

**Affiliations:** Department of Neurological Rehabilitation, Zhengzhou Central Hospital Affiliated to Zhengzhou University, Zhengzhou, China

**Keywords:** functional disability, chronic low back pain, balance, age, emotion

## Abstract

**Introduction:**

Postural balance is impaired in patients with chronic low back pain (CLBP). In addition, the swaying velocity can be aﬀected by low back pain (LBP) dysfunction. However, the extent to which the dysfunction aﬀects postural balance in CLBP patients remains unclear. Therefore, this study aimed to investigate the eﬀect of LBP-related disability on postural balance among CLBP patients and determine factors associated with postural balance impairments.

**Methods:**

Participants with CLBP were recruited and instructed to complete the one-leg stance and Y-balance test. Moreover, they were divided into two subgroups (i.e., low and medium to high LBP-related disability groups) to compare the diﬀerence in postural balance based on the degree of LBP-related disability measured by the Roland Morris Disability Questionnaire. The relationships between postural balance and negative emotions as well as LBP characteristics were determined using the Spearman correlations.

**Results:**

A total of 49 participants with low LBP-related disabilities and 33 participants with medium to high LBP-related disabilities participated in the study. Compared to the medium to high LBP-related disability group, patients in the low LBP-related disability group performed better in one-leg stance on the left leg (*z* = -2.081, *p* = 0.037). For Y-balance test, patients in the low LBP-related disability group also had greater normalized values of left leg reach in posteromedial (*t* = 2.108, *p* = 0.038) direction and composite score (*t* = 2.261, *p* = 0.026) and of right leg reach in posteromedial (*t* = 2.185, *p* = 0.032), and posterolateral (*t* = 2.137, *p* = 0.036) directions as well as composite score (*t* = 2.258, *p* = 0.027). Factors associated with postural balance impairments were also revealed, such as anxiety, depression, and fear avoidance belief.

**Discussion:**

The greater the dysfunction degree, the worse the CLBP patient’s postural balance impairment. Negative emotions could also be considered contributing factors for postural balance impairments.

## 1. Introduction

Low back pain (LBP) is one of the most frequently occurring musculoskeletal complaints with a lifetime prevalence of up to 80% (1, 2). Although in most cases, the disease is self-limited and naturally resolves within 6 weeks, approximately 20% of cases may progress to chronic low back pain (CLBP), thereby resulting in greater burdens on health and economy (3). However, CLBP is difficult to treat and is associated with various mechanical changes (4). After taking age into account as a major determinant of balance, LBP may still explain 9% of the variance in balance (5).

Postural balance is the ability to maintain or restore body balance under static or dynamic conditions (6), which requires stable central nervous system function and good sensory and motor system function. Postural balance in patients with CLBP may be impaired by central and/or peripheral mechanisms. Constant interaction between central and peripheral mechanisms is required to maintain balance. Peripheral mechanisms of balance include (1) somatosensory systems (e.g., receptors in joints, muscles, and ligaments) that provide input regarding joint position and force, (2) the visual system, which provides input about the environment, and (3) the vestibular system, which provides input regarding angular velocity and linear acceleration. The central nervous system integrates peripheral inputs and controls appropriate muscular responses to maintain balance. If any of these components are impaired, postural balance impairment may occur. The core basis of CLBP is a chronic injury to the muscles of the low back, accompanied by changes in the nervous system (7). For example, Henry et al. (8) reported that individuals with CLBP have abnormal automatic postural coordination, which indicates altered neuromuscular control (8). And Yu et al. (9) found that individuals with CLBP had lower cerebellar gray matter density which is essential for balance control (9).

Thus, optimal postural balance is necessary to perform normal activities of daily living. Although balance assessments have been performed with expensive laboratory equipment, such laboratory equipment is impractical in a clinical setting (10–12). One-leg stance (OLS) and Y-balance test (YBT) are available and inexpensive to measure static and dynamic balance, which have high intra- and inter-rater reliability and can be easily implemented in clinical practice (12, 13).

When compared to healthy controls, there is an impaired performance in OLS and YBT in those with CLBP, which may affect spinal stability (14). Impaired postural balance may increase the compression load on the spine, thereby resulting in the risk of future deterioration (15). In addition, CLBP patients might modify their motor control strategies in an attempt to prevent further pain (16, 17). Postural balance may be influenced by other factors, such as age, impaired proprioception, muscle strength, and coordination (16, 18). As there is a lack of evidence on the effect of negative emotions on postural balance in those with CLBP (16), their relationships remain to be determined.

Furthermore, functional disability may be a potential contributing factor to postural strategies adopted by CLBP patients (19). A Brazilian epidemiological study has shown that 27.7 and 22.7% of CLBP patients had LBP-related disabilities and changes in postural balance, respectively (20). Brech et al. (4) found that the degree of LBP-related disability is negatively correlated with the swaying velocity of OLS among women with CLBP (4), which means CLBP women with greater dysfunction have better postural balance. This unexpected result may be due to the subjects’ compensation and the number and type of subjects included (10 women). Thus, whether the effect of LBP-related disability on postural balance would have similar results in the general CLBP population remains unclear.

Assessment of balance is an important management aspect of CLBP, providing useful clinical information that could be incorporated into rehabilitation programs. Thus, this study aims to explore the effect of LBP-related disability on postural balance among patients with CLBP, and determine the factors (e.g., negative emotions and LBP characteristics) related to postural balance. The first hypothesis of this study is that participants with greater LBP-related disability would have worse balance performance in OLS and YBT. The second hypothesis is that negative emotions (e.g., anxiety, depression, and fear avoidance belief) and LBP characteristics would be negatively correlated with postural balance.

## 2. Materials and methods

### 2.1. Participants

CLBP subjects were recruited in a local community through posted advertisements. Inclusion criteria were pain in the lumbar and sacroiliac joint regions that had lasted more than 12 weeks before the test and an average pain intensity score of ≥2/10 on the numerical rating scale (NRS) in the past week. Alshehre et al. (12) found a moderate effect for the Y balance test in young adults with CLBP of NRS ≥ 2/10 (12). Meanwhile, exclusion criteria were patients who reported current pregnancy, signs of nerve root compression, serious spinal conditions (e.g., tumor, infection, or fracture), specific balance issues (e.g., vestibular apparatus problem or uncorrected visual disturbances), history of surgery to the lower extremity or spine, or pain in the joints of the lower extremities. In addition, none of the participants had been treated with CLBP in the past 12 weeks. This study was approved by the Ethics Committee of Zhengzhou Central Hospital Affiliated to Zhengzhou University (No.: 201966).

Once participants were determined to be eligible for the study, their demographic and clinical characteristics were collected, including age, sex, height, weight, and body mass index (BMI), as well as leg length (LL), duration, and location of LBP. LL is the distance between the anterior superior iliac spine and the ipsilateral medial malleolus at supine position (12, 21). Furthermore, participants rated their current pain and average pain intensity in the past week using the NRS. Participants also completed the Roland Morris Disability Questionnaire (RMDQ) to evaluate LBP-related disability, the Self-Rating Anxiety Scale (SAS) to evaluate anxiety in the last week, the Self-Rating Depression Scale (SDS) to evaluate depression in the last week, and the Fear-Avoidance Beliefs Questionnaire for physical activities (FABQp) and work activities (FABQw) to evaluate the fear-avoidance level.

As a widely employed scale with good validity and reliability, RMDQ can allow quantification and further classification of LBP by the extent of its impact on physical function (19). Although cutoff points of RMDQ have not been established clearly, Kimachi et al. (19) found that the level of dysfunction based on RMDQ was associated with an increased risk of falls in the elderly, and the incidence of any fall over the past year was 27.8% of those with low LBP-related disability (RMDQ = 1–5) and 37.6% of those with medium to high level of LBP-related disability (RMDQ = 6–24) (19). Thus, in the present study, participants were divided into two subgroups based on the degree of LBP-related disability measured by RMDQ to compare the difference in postural balance: low (RMDQ = 1–5) and medium to high LBP-related disability (RMDQ = 6–24).

### 2.2. Procedures

Herein, subjects were asked to perform the OLS to assess their static postural balance (22). During the test, subjects were asked to stand on one leg on a force platform with bare feet and eyes closed. The test ended when the non-standing foot touched the floor or the standing leg, and a timer was used to record the duration. The order of one-leg stance on the left (OLS-L) and right (OLS-R) legs was randomized with random numbers table to minimize the order effect. Subjects practiced thrice on each leg for familiarization. Then, they completed three trials for each leg with the mean value for analysis. There was a 30 s break between trials to avoid fatigue.

**Figure 1:**
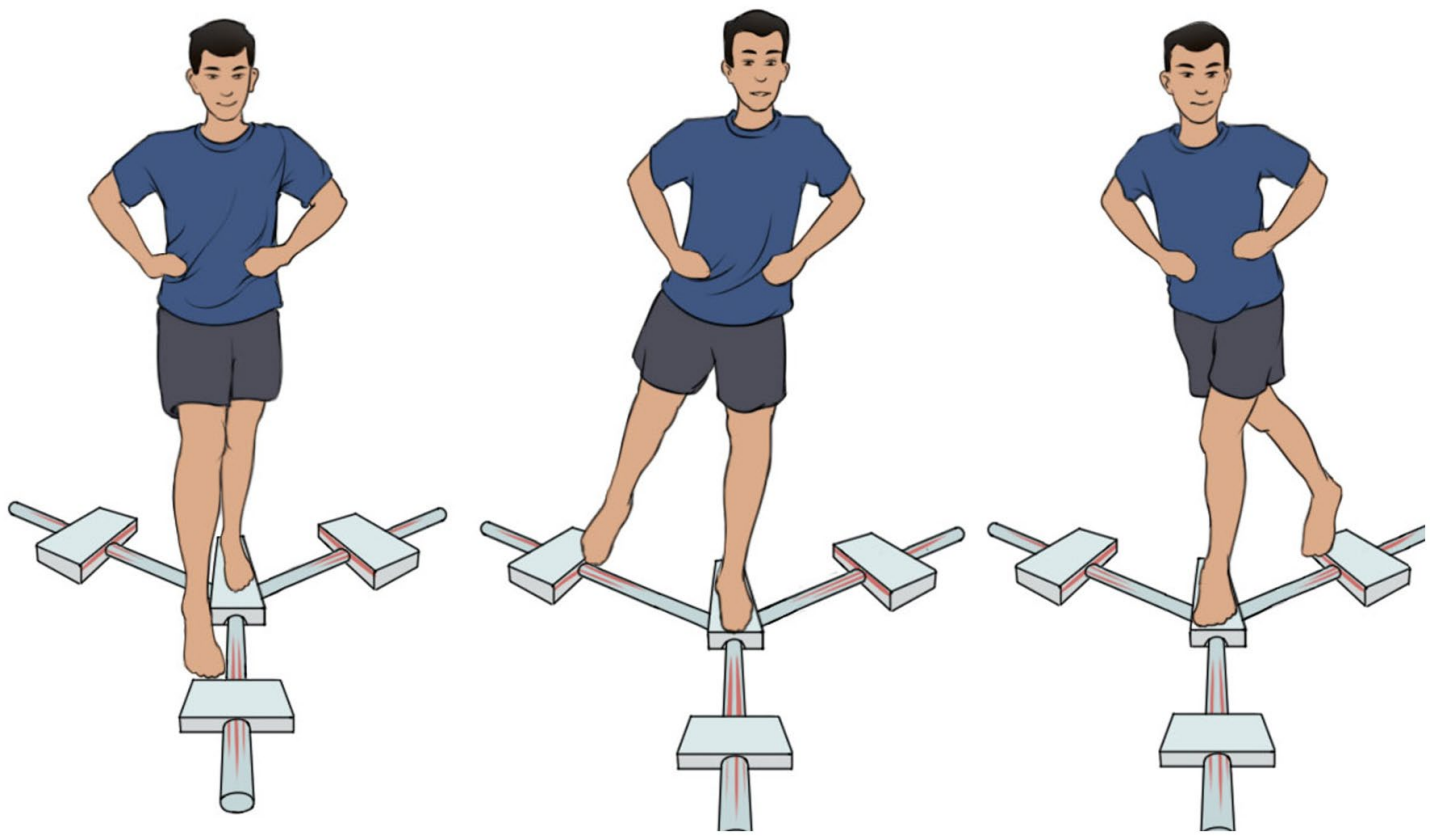
The Y balance test in the anterior (left part), posterolateral (middle part), and posteromedial (right part) reach directions. Here’s an example of the right leg reach.

Afterward, subjects were instructed to complete YBT correctly to evaluate their dynamic balance (12). The test involves maximal reaching distances in anterior (AT), posterolateral (PL), and posteromedial (PM) directions from the central stance platform at an angle of 120° (Figure 1). During the test, participants were instructed to keep the standing leg on the central stance platform barefooted, and use another bare foot to slide and reach the indicators in three directions as far as possible, and then return to their initial standing position while maintaining their balance. Moreover, the testing orders of the reaching directions and legs were randomized with random numbers table to minimize the order effect. Subjects practiced thrice in each direction for familiarization. Then, they completed three trials in each direction with the mean value for analysis. There were 10 s breaks between trials and 30 s breaks between reach directions and between legs to minimize fatigue. The outcome measures of interest were the distances to be reached in the three directions, the composite score (CS), and the normalized reach. The composite score was calculated as [(AT ± PM ± PL)/(3 × LL)]. Normalized reach was calculated as [(reach distance/LL) × 100%] (12).

### 2.3. Statistical analysis

According to Alshehre et al. (12), a calculation by G*Power software (v3.1) showed that at least 62 participants (31 per group) were required to detect a moderate effect of Y balance test (power = 0.80, *α* = 0.05) (12). All data were analyzed using the SPSS software (v23.0). The statistical significance level was set at *p* < 0.05. The Chi-square test was utilized to explore the significant differences in categorical variables between the groups. The Kolmogorov–Smirnov test was used to analyze whether or not the continuous variables were normally distributed. To test the first hypothesis, independent T-tests (for normally distributed variables) and Mann-Whiney U-tests (for non-normally distributed variables) were utilized to explore the balance performance between low and medium to high LBP-related disability groups. To address the second hypothesis, Spearman correlations were applied to describe the relationships between postural balance and its influencing factors.

## 3. Results

### 3.1. Participants’ characteristics

A total of 49 participants with low LBP-related disabilities and 33 participants with medium to high LBP-related disabilities participated in this study. Participants’ characteristics of both groups are summarized in Table 1. The low LBP-related disability group consisted of 19 men and 30 women with a mean age of 26.16 ± 5.47 years, whereas the medium to high LBP-related disability group consisted of 11 men and 22 women with a mean age of 28.42 ± 6.50 years. However, demographic characteristics of age, gender, height, weight, BMI, and LL were not significantly different between groups (*p* > 0.05). Moreover, no significant clinical differences were observed in LBP duration, pain location, pain intensity, and anxiety between groups (*p* > 0.05). However, significant differences were found in depression and fear-avoidance levels (including total score, FABQp, and FABQw), which were higher in the medium to high LBP-related disability group (*p* < 0.05).

**Table 1:** Demographic and clinical characteristics of the participants.

	Total (*n* = 82)	LBP-related disability			
		Low LBP-related disability (*n* = 49)	Medium to high LBP-related disability (*n* = 33)	*χ*^2^/*t*/*z*	*p*
Sex ratio (male/female)	30/52	19/30	11/22	0.252	0.616
Age (years)	27.07 ± 5.97	26.16 ± 5.47	28.42 ± 6.50	−1.580^a^	0.114
Height (cm)	166.80 ± 8.09	166.98 ± 7.51	166.52 ± 9.01	0.251	0.802
Weight (kg)	63.52 ± 11.95	63.24 ± 10.36	63.93 ± 14.14	−0.241	0.810
BMI (kg/m^2^)	22.59 ± 3.32	22.40 ± 2.75	22.88 ± 4.05	−0.639	0.525
Left leg length (cm)	84.86 ± 5.08	84.71 ± 4.41	85.08 ± 6.01	−0.142^a^	0.887
Right leg length (cm)	84.72 ± 5.11	84.57 ± 4.49	84.93 ± 5.97	−0.204^a^	0.839
LBP duration (years)	4.89 ± 2.44	5.09 ± 2.29	4.58 ± 2.64	−1.823^a^	0.068
Pain location					
Left side only	14	6	8	4.959	0.175
Right side only	21	15	6		
Both sides	31	22	9		
Median position	23	12	11		
Pain (1–10 NRS)					
Current pain	2.65 ± 1.49	2.45 ± 1.34	2.94 ± 1.68	−1.383^a^	0.167
Average pain	3.39 ± 1.37	3.20 ± 1.31	3.67 ± 1.43	−1.358^a^	0.175
SAS	41.99 ± 8.51	40.80 ± 7.38	43.76 ± 9.80	−1.270^a^	0.204
SDS	40.80 ± 9.82	38.37 ± 7.89	44.42 ± 11.32	−2.249^a^	**0.025**
FABQ-total	36.18 ± 11.30	32.69 ± 11.73	41.36 ± 8.39	−3.658	**<0.001**
FABQp	11.62 ± 5.14	10.69 ± 5.49	13.00 ± 4.29	−2.107^a^	**0.035**
FABQw	24.56 ± 8.69	22.00 ± 8.86	28.36 ± 6.98	−3.465	**0.001**

The participants’ characteristics are presented as mean ± standard deviation, ratio, or frequency. Low LBP-related disability, RMDQ = 1–5; Medium to high LBP-related disability, RMDQ = 6–24; BMI, body mass index; FABQ, fear avoidance beliefs questionnaire; FABQp, fear avoidance beliefs questionnaire for physical activities; FABQw, fear avoidance beliefs questionnaire for work activities; NRS, numeric rating scale; RMDQ, Roland Morris disability questionnaire.

a Mann–Whiney U-tests. The bold value means *p*<0.05.

### 3.2. Static balance

Table 2 displays the OLS scores for both groups. The OLS-L score was significantly higher in the low LBP-related disability group (*z* = −2.081, *p* = 0.037), whereas the OLS-R had no significant difference between groups (*t* = 1.429, *p* = 0.157).

**Table 2:** Comparison of OLS scores between groups.

	Total (*n* = 82)	LBP-related disability			
		Low LBP-related disability (*n* = 49)	Medium to high LBP-related disability (*n* = 33)	*t*/*z*	*p*
OLS-L	51.96 ± 34.63	58.35 ± 35.74	42.48 ± 31.05	−2.081^a^	**0.037**
OLS-R	54.23 ± 32.30	58.39 ± 30.80	48.06 ± 33.93	1.429	0.157

Data are presented as mean ± standard deviation. Low LBP-related disability, RMDQ = 1–5; Medium to high LBP-related disability, RMDQ = 6–24; OLS-L, one-leg stance on the left leg; OLS-R, one-leg stance on the right leg; RMDQ, Roland Morris disability questionnaire.

a Mann–Whiney U-tests. The bold value means *p*<0.05.

### 3.3. Dynamic balance

Table 3 presents the YBT scores for both groups. No significant differences were found in the absolute reaching distances in all directions and composite scores between groups. However, concerning the normalized reach, the low LBP-related disability group had significant scores in PM (*p* = 0.038) and CS (*p* = 0.026) of the left leg reach, PM (*p* = 0.032), PL (*p* = 0.036), and CS (*p* = 0.027) of the right leg reach as compared to those of the medium to high LBP-related disability group.

**Table 3:** Comparison of YBT scores between groups.

	Total (*n* = 82)	LBP-related disability			
		Low LBP-related disability (*n* = 49)	Medium to high LBP-related disability (*n* = 33)	*t*/*z*	*p*
*Left leg reach*					
AT (cm)	62.17 ± 9.33	63.35 ± 10.62	60.43 ± 6.79	−0.989^a^	0.323
PM (cm)	97.30 ± 15.26	99.75 ± 16.89	93.67 ± 11.78	1.793	0.077
PL (cm)	98.72 ± 12.41	100.41 ± 13.93	96.22 ± 9.40	1.627	0.108
CS (cm)	86.06 ± 10.76	87.83 ± 11.92	83.44 ± 8.25	1.974	0.052
*Right leg reach*					
AT (cm)	63.18 ± 11.26	64.37 ± 13.28	61.41 ± 7.14	−0.648^a^	0.517
PM (cm)	98.09 ± 11.36	99.95 ± 11.54	95.33 ± 10.66	1.831	0.071
PL (cm)	99.16 ± 11.87	101.01 ± 13.33	96.41 ± 8.78	1.884	0.063
CS (cm)	86.81 ± 9.88	88.44 ± 10.91	84.39 ± 7.65	1.976	0.052
*Left leg reach*					
AT (% LL)	73.26 ± 9.96	74.70 ± 11.14	71.13 ± 7.53	1.611	0.111
PM (% LL)	114.52 ± 15.38	117.40 ± 16.44	110.25 ± 12.74	2.108	**0.038**
PL (% LL)	116.35 ± 13.08	118.46 ± 14.66	113.22 ± 9.69	1.949	0.055
CS (% LL)	101.38 ± 10.67	103.51 ± 11.47	98.21 ± 8.58	2.261	**0.026**
*Right leg reach*					
AT (% LL)	74.58 ± 12.25	75.97 ± 14.13	72.52 ± 8.54	1.254	0.213
PM (% LL)	115.83 ± 11.72	118.10 ± 11.24	112.46 ± 11.77	2.185	**0.032**
PL (% LL)	117.02 ± 11.90	119.27 ± 13.09	113.67 ± 9.05	2.137	**0.036**
CS (% LL)	102.48 ± 9.88	104.45 ± 10.39	99.55 ± 8.39	2.258	**0.027**

Data are presented as mean ± standard deviation. First, absolute values (cm) are shown followed by normalized values (% LL). Low LBP-related disability, RMDQ = 1–5; Medium to high LBP-related disability, RMDQ = 6–24; AT, anterior; CS, composite score; LL, leg length; PL, posterolateral; PM, posteromedial; RMDQ, Roland Morris disability questionnaire.

a Mann–Whiney U-tests. The bold value means *p*<0.05.

### 3.4. Correlation analysis

Table 4 shows the relationships between balance and demographic and clinical characteristics. The normalized values of left and right leg reach were selected for analysis. Age was negatively correlated with both legs’ reach in multiple directions and OLS-R score. Duration of LBP was positively correlated with left leg reach in the AT direction (*r* = 0.287, *p* = 0.005). As for the pain intensity, current pain is positively related to the left leg reach in the AT direction (*r* = 0.228, *p* = 0.020). SAS showed an inverse correlation with left leg reach in the PM direction (*r* = −0.186, *p* = 0.047) and CS (*r* = −0.223, *p* = 0.022), and SDS was negatively related to left leg reach in the PL direction (*r* = −0.193, *p* = 0.041) and CS (*r* = −0.208, *p* = 0.030), and right leg reach in the AT direction (*r* = −0.205, *p* = 0.032), and CS (*r* = −0.210, *p* = 0.029). Moreover, fear-avoidance levels (including total score, FABQp, and FABQw) were negatively correlated with both legs reaching in multiple directions, and FABQp presented an inverse correlation with the OLS-R score (*r* = −0.215, *p* = 0.026).

**Table 4:** The correlation between postural balance and demographic and clinical characteristics.

	Left leg reach				Right leg reach				OLS-L	OLS-R
	Left AT	Left PM	Left PL	Left CS	Right AT	Right PM	Right PL	Right CS		
Age	−0.057 (0.305)	**−0.202 (0.035)**	**−0.198 (0.037)**	**−0.224 (0.021)**	**−0.192 (0.042)**	−0.026 (0.408)	**−0.238 (0.016)**	**−0.213 (0.027)**	−0.167 (0.067)	**−0.211 (0.029)**
BMI	−0.099 (0.189)	0.162 (0.073)	0.147 (0.095)	0.094 (0.201)	−0.060 (0.295)	0.152 (0.087)	0.161 (0.074)	0.107 (0.168)	−0.161 (0.074)	0.004 (0.486)
LBP Duration	**0.287 (0.005)**	0.109 (0.165)	0.104 (0.176)	0.155 (0.082)	0.072 (0.259)	0.085 (0.223)	0.002 (0.492)	0.061 (0.294)	0.003 (0.490)	0.055 (0.312)
Current Pain	**0.228 (0.020)**	0.108 (0.166)	0.151 (0.088)	0.168 (0.065)	0.183 (0.050)	−0.034 (0.382)	0.100 (0.186)	0.137 (0.110)	0.064 (0.284)	0.162 (0.073)
Average Pain	0.147 (0.093)	−0.035 (0.378)	0.048 (0.335)	0.028 (0.400)	0.024 (0.415)	0.009 (0.468)	−0.038 (0.369)	0.036 (0.372)	0.050 (0.327)	0.097 (0.194)
SAS	−0.153 (0.085)	**−0.186 (0.047)**	−0.179 (0.054)	**−0.223 (0.022)**	−0.173 (0.061)	−0.143 (0.099)	−0.092 (0.206)	−0.166 (0.069)	−0.068 (0.271)	−0.173 (0.060)
SDS	−0.130 (0.122)	−0.152 (0.086)	**−0.193 (0.041)**	**−0.208 (0.030)**	**−0.205 (0.032)**	−0.167 (0.067)	−0.146 (0.095)	**−0.210 (0.029)**	−0.126 (0.130)	−0.106 (0.171)
FABQ-Total	**−0.213 (0.028)**	**−0.245 (0.013)**	**−0.206 (0.032)**	**−0.248 (0.012)**	−0.180 (0.053)	−0.113 (0.157)	**−0.184 (0.049)**	−0.168 (0.065)	−0.068 (0.271)	−0.015 (0.446)
FABQp	−0.144 (0.098)	−0.179 (0.054)	−0.135 (0.113)	**−0.192 (0.042)**	−0.119 (0.144)	−0.075 (0.253)	**−0.185 (0.048)**	−0.172 (0.061)	−0.145 (0.097)	**−0.215 (0.026)**
FABQw	−0.179 (0.053)	**−0.204 (0.033)**	**−0.201 (0.035)**	**−0.200 (0.036)**	−0.159 (0.076)	−0.114 (0.154)	−0.135 (0.113)	−0.118 (0.146)	0.069 (0.268)	0.138 (0.109)

Data are correlation coefficient (*p* value). Left leg reach and right leg reach are analyzed with normalized values (% LL). AT, anterior; CS, composite score; LL, leg length; PL, posterolateral; PM, posteromedial; OLS-L, one-leg stance on the left leg; OLS-R, one-leg stance on the right leg; FABQ, fear avoidance beliefs questionnaire; FABQp, fear avoidance beliefs questionnaire for physical activities; FABQw, fear avoidance beliefs questionnaire for work activities. One-tailed Spearman analysis was used. The bold value means *p*<0.05.

## 4. Discussion

This study evaluated the associations between disability and postural balance in patients with CLBP. We divided 82 patients into low and medium to high LBP-related disability groups. We found that patients in the low LBP-related disability group performed better in OLS and YBT than the medium to high LBP-related disability group. Factors that influence postural balance, such as age, LBP duration, pain intensity, and negative emotions were also revealed.

### 4.1. Effects of disability on postural balance

In our study, CLBP patients with medium to high dysfunctions were less likely to maintain their balance in OLS-L as compared to those with low dysfunctions, thereby demonstrating a different outcome to the findings of Tsigkanos et al. (23) which indicates that CLBP participants had worse performance in the OLS-R (23). It could be because of differences in the included subjects. Tsigkanos et al. (23) included patients with a higher mean age and no assessment of dysfunction. Thus, the finding of OLS partially supports the original hypothesis that the more severe the LBP-related disability, the worse the static balance.

Although no significant differences were observed in the absolute values of the YBT reaching distance between groups, the normalized reach distances in PM, PL, and CS were significantly higher in the low LBP-related disability group as compared to the medium to high LBP-related disability group. This may be because highly dysfunctional subjects may be accompanied by functional impairments in other body parts, such as lower limbs. For example, poor balance in the posterior direction predicted ankle sprains (24, 25). The YBT composite score showed that the CLBP participants’ ability to perform multiplanar motion decreased with the increase of the dysfunction (26). However, no differences were observed in the AT direction, which was similar to the findings of Hooper et al. (2016) (21). It may be due to the vision that compensates for any somatosensory and vestibular issues during the reaching in AT direction (21, 27). In PM and PL directions, the lower limbs are placed out of sight behind the body, thereby relying more on somatosensory and vestibular sense to complete the balance tasks. That is, the increase in difficulty may further expose deficiencies in dynamic balance in CLBP patients with higher dysfunction, again supporting our original hypothesis.

Perfect posture balance requires stable nervous system function. Normal nervous functions base on normal anatomical structures such as cerebellum, spinal cord, and peripheral nerves, which are essential for the processing of balance information and the timely and effective balance response. Although this paper cannot provide evidence of neural mechanisms, CLBP has been shown to cause neurological changes, such as cerebellar atrophy, white matter damage, and decreased sensory perception (9). And postural balance is related to functional parameters such as muscle strength, flexibility, muscle control, and proprioception (23). Thus, postural balance impairments in CLBP patients implicate not only abnormal lumbar muscle conditions but also underlying neurological changes leading to neural inefficiency, which can be further investigated by combining neuroimaging techniques such as functional near-infrared spectroscopy.

### 4.2. Other factors related to postural balance

Our findings of a significant negative correlation between age and performance of YBT and OLS are consistent with previous research (22, 23). Postural balance deteriorates with age possibly caused by biological changes (e.g., neuromusculoskeletal alterations, mobility issues, and sensory-motor deficits) (22, 28). Particularly, impaired postural balance increases the risk of falls in older CLBP patients (29). In addition, BMI is unlikely to affect postural balance, which is consistent with da Silva et al. (2019) (22). Interestingly, LBP duration and current pain intensity were found to be significantly positively correlated with the left leg reaching distance (normalized value) in AT direction. However, Brech et al. (4) found that the lumbar pain intensity and frequency were not correlated with postural balance in women with CLBP (4), which may be because the included participants were younger and their compensatory capacity was high (30). Moreover, we found that the scores of depression and fear avoidance belief (except for anxiety) were higher in the medium to high LBP-related disability group than in the low LBP-related disability group. Moreover, anxiety, depression, and fear avoidance belief were negatively related to YBT performance in multiple directions and only the FABQp score presented a significant inverse correlation with the OLS-R score. Recently, researchers have proposed that negative emotions could affect motor control. For example, anxiety and depression disrupt sensory integration and cognitive interaction (31, 32), requiring greater attention demands to maintain balance. However, although there is a lack of evidence on the effect of pain-related anxiety and depression on postural balance, it is theoretically possible that anxiety and depression could affect postural balance (16). Furthermore, CLBP patients with high fear-avoidance beliefs were not only vigilant but also significantly restricted their range of motion during lumbar flexion and extension (18). Fear may also disrupt the automaticity of neural control pathways, leading to deficits in trunk motor control and increased trunk variability during maintaining balance, which may also increase the risk of LBP (33). Dynamic balance is a more difficult condition than static balance, which makes it easier to reveal the relationship between negative emotions and dynamic balance ability in YBT (34).

### 4.3. Limitations

However, there are still some limitations despite the findings in this paper. First, we included only young subjects and did not include subjects from other age groups, such as elderly patients with CLBP. And we lack evidence from healthy controls, i.e., healthy young adults with no disabilities. Thus, future studies should study a wider age range of subjects and include healthy controls to enhance the applicability of the findings in this paper. Second, this is a cross-sectional study. Thus, longitudinal research can be further carried out to investigate the effects of LBP-related disability and other factors on postural balance. Furthermore, this paper is limited to behavioral results and lacks neuroimaging evidence, so neuroimaging techniques should be further used to explore the neural mechanism of the effect of LBP-related disability on balance impairments.

## 5. Conclusion

The greater the dysfunction degree, the worse the CLBP patient’s postural balance. In addition, age, and negative emotions (i.e., anxiety, depression, and fear avoidance belief) could be considered contributing factors for postural balance impairments. Given that postural balance is critical for CLBP patients’ daily living, it should be thoroughly evaluated and integrated into LBP management.

## Data availability statement

The raw data supporting the conclusions of this article will be made available by the authors, without undue reservation.

## Ethics statement

The studies involving human participants were reviewed and approved by the Ethics Committee of Zhengzhou Central Hospital Affiliated to Zhengzhou University. The patients/participants provided their written informed consent to participate in this study.

## Author contributions

PS, KL, XY, ZW, and YY conceptualized this study and contributed to revising and approving the final version of the manuscript. PS and KL contributed to collecting data. Analyzing the data and drafting the manuscript were by PS. All authors contributed to the article and approved the submitted version.

## Funding

This study was supported by the General Project of Medical Science and Technology Project of Health Department of Henan Province (2018020808).

## Conflict of interest

The authors declare that the research was conducted in the absence of any commercial or financial relationships that could be construed as a potential conflict of interest.

## Publisher’s note

All claims expressed in this article are solely those of the authors and do not necessarily represent those of their affiliated organizations, or those of the publisher, the editors and the reviewers. Any product that may be evaluated in this article, or claim that may be made by its manufacturer, is not guaranteed or endorsed by the publisher.
